# The Influence of 2′-Deoxyguanosine Lesions on the Electronic Properties of ^OXO^G:::C Base Pairs in Ds-DNA: A Comparative Analysis of Theoretical Studies

**DOI:** 10.3390/molecules29163756

**Published:** 2024-08-08

**Authors:** Boleslaw T. Karwowski

**Affiliations:** DNA Damage Laboratory of Food Science Department, Faculty of Pharmacy, Medical University of Lodz, ul. Muszynskiego 1, 90-151 Lodz, Poland; boleslaw.karwowski@umed.lodz.pl

**Keywords:** DNA damage, 7,8-dihydro-8-oxo-2′-deoxyguanosine, electron-hole transfer, DFT, glycosylases

## Abstract

DNA is continuously exposed to a variety of harmful factors, which, on the one hand, can force undesirable processes such as ageing, carcinogenesis and mutagenesis, while on the other hand, can accelerate evolutionary changes. Of all the canonical nucleosides, 2′-deoxyguanosine (dG) exhibits the lowest ionization potential, making it particularly prone to the one-electron oxidizing process. The most abundant type of nucleobase damage is constituted by 7,8-dihydro-8-oxo-2′-deoxyguanosine (^OXO^dG), with an oxidation potential that is 0.56 V lower than that of canonical dG. All this has led to ^OXO^dG, as an isolated lesion, being perceived as a sink for radical cations in the genome. In this paper, a comparative analysis of the electronic properties of an ^OXO^GC base pair within the context of a clustered DNA lesion (CDL) has been conducted. It is based on previous DFT studies that were carried out at the M06-2x/6-31++G** level of theory in non-equilibrated and equilibrated condensed phases. The results of the comparative analysis presented here reveal the following: (A) The ionization potentials of ^OXO^G_4_C_2_ were largely unaffected by a second lesion. (B) The positive charge and spin were found predominantly on the ^OXO^G_4_C_2_ moiety. (C) The electron-hole transfers A_3_T_3_→G_4_C_2_ and G_4_C_2_←A_5_T_1_ were found in the Marcus inverted region and were resistant to the presence of a second DNA lesion in close proximity. It can therefore be reasonably postulated that ^OXO^GC becomes the sink for a radical cation migrating through the double helix, irrespective of the presence of other 2′-deoxyguanosine lesions in the CDL structure.

## 1. Introduction

The seed of life is stored in the genome of every living organism. DNA strands are arranged in a sequence of nucleotides including four canonical nucleosides interconnected by phosphodiester which act as building “blocks” [1,2,3]. However, this encoded information is continuously exposed to a variety of harmful chemical and physical factors [4]. The stability of DNA faces constant threats from a number of endocellular and exocellular sources, including oxygen and nitrogen species, reactive lipid metabolites, xenobiotics, pollution and various types of radiation such as UV, X-ray, gamma (γ), beta (β) and alpha (α) radiation [5,6,7]. The activities of the latter physical factors can also lead to water radiolysis, with the generation of hydroxyl radicals (●OH) via the following reaction scheme: H_2_O + γ→H_2_O^+^ + e^−^ → H^+^ + ●OH. In the context of anticancer therapy, induction of the instability of genetic information is highly significant [8]. The type and amount of DNA damage formed depends on the source of the damaging factors and conditions of the endocellular environment (normoxic or hypoxic) [9,10].

**Figure 1:**
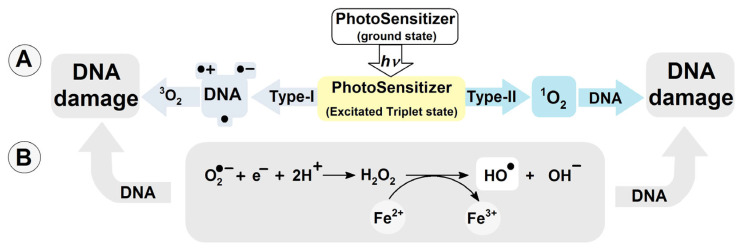
(**A**) DNA damage induction by photosensitizers Type-I and II; (**B**) hydroxyl radical formation via Haber–Weiss reaction catalyzed by transition metal ions.

To date, more than 80 kinds of DNA damage have been identified [11]. They are substrates for different DNA repair systems and include, depending on their chemical structure, base excision repair (BER), global genome (GG) and transcription-coupled (TG) nucleotide excision repair (NER), homologous recombination (HR), non-homologous end joining (NHEJ), and mismatch repair (MR) [12,13]. The most common of these is the BER system, which removes simple isolated lesions like apurinic/apyrimidinic (AP) sites, single-strand breaks (SSBs), and oxidized nucleobases [14,15]. Of all the nucleobases, guanine (Gua) exhibits the lowest ionization potential. The oxidation potentials of adenine, thymine, cytosine and guanine have been determined as follows [V]: 1.97, 2.11, 2.14 and 1.49 [16], while the midpoint potentials (*E*_7_) are, respectively: 1.29 (dG(-H)^●^/dG), 1.42 (dA(-H)^●^/dA), 1.70 (dT(-H)^●^/dT), 1.60 (dC(-H)^●^/dC) at neutral pH [17]. Moreover, guanosine reacts exclusively with singlet oxygen ^1^O_2_ (with reference to the ^1^*Δ* state), forming 7,8-dihydro-8-oxo-2′-deoxyguanosine. Note that the ground state of oxygen is triplet ^3^O_2_ [18]. Cytosolic singlet oxygen ^1^O_2_ can result from type-II photosensitization, such as that induced by photoreactive drugs (*λ* > 360 nm) with a triplet–triplet energy transfer to oxygen [19]. Type-I photosensitizers can react with nucleic acid moieties, leading to hydrogen or electron transfer, culminating in the formation of neutral radicals or radical ions (Figure 1A) [20]. Also, type-I photosensitizers can produce O_2_^●−^ and coupled HO_2_^●^ via electron donation or charge transfer to molecular oxygen [21]. It should be noted that reactive oxygen species (ROS) like H_2_O_2_, and O_2_^●−^ are suitable substrates for the transition metal-catalyzed Haber–Weiss reaction, acting as a source of the highly reactive hydroxyl radical (Figure 1B) [22,23]. ●OH can modify nucleosides and nucleotides either by hydrogen atom abstraction or an addition reaction to the unsaturated bonds with a diffusion-controlled rate constant *k* of approximately 2.5 × 10^8^ M^−1^s^−1^ [24].

In the nucleus or mitochondria, DNA adopts a double helix structure, formed by two complementary counter-rotating strands. The stability of ds-DNA depends equally on the hydrogen bond energy (*E_HB_*) and *π*–*π* stacking interaction energy (*E_ST_*) between the proximal base pairs [25]. However, both factors are sensitive to structural changes. This unique structure allows charge transfer through the double helix at a distance of at least 200 [Å], much like a graphene nanowire [26,27]. The radical cation (●+) being induced, within the double helix, by, for example, photosensitizers or UV activity, can migrate through ds-DNA until it settles at the predisposed site [28,29]. It has been shown that areas rich in guanine becomes the sink for the radical cation. Additionally, the ionization potential (IP) decreases as the number of guanines increases: G > GG > GGG [30]. The details of charge transfer between guanosines in ds-oligo have been discussed in reference [31,32].

**Figure 2:**
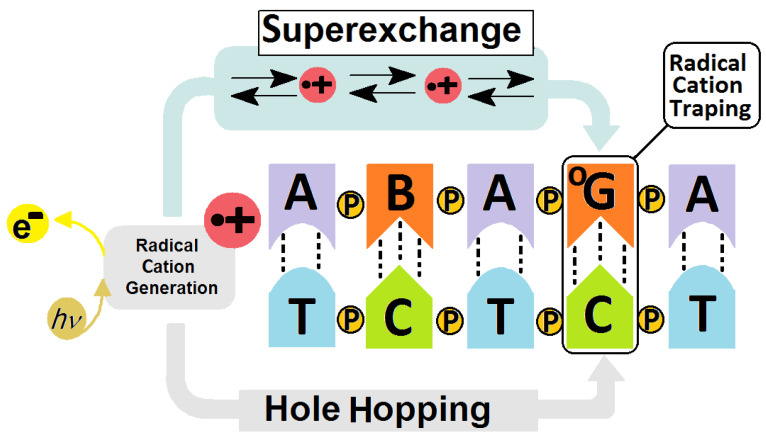
Graphical representation of radical cation induction and transfer in a DNA double helix showing the schematic difference between the Hole Hopping (long distance) and Superexchange (short distance) mechanisms of charge migration. B—noncanonical nucleoside, ^O^G—7,8-dihydro-8-oxo-2′deoxyguanosine, P—phosphodiester internucleotide bond, e^−^—electron, *h*—Planck constant, *ν*—frequency [16,31,32].

**Figure 3:**
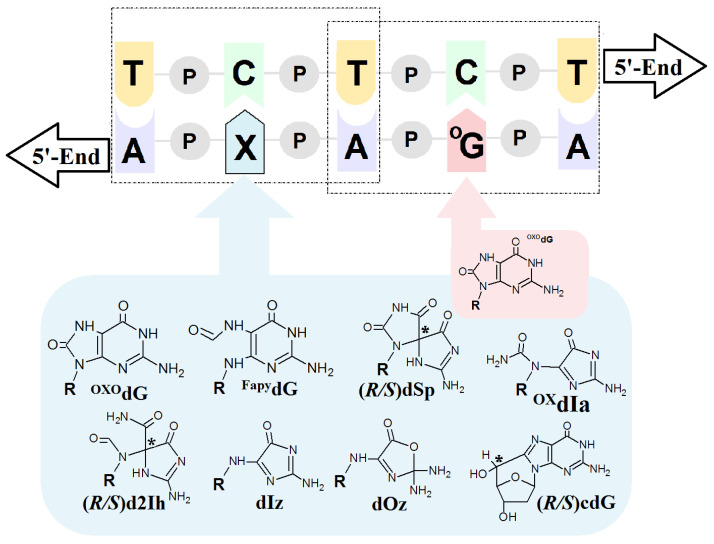
Graphical representation of a model ds-DNA sequence indicating the distribution of selected lesions with their structural graphical representation. **^OXO^dG**: 7,-8-dihydro-8-oxo-guanine; **^Fapy^dG**: 2,6-diamino-4-hydroxy-5-formamidopyrimidine; **dSp**: spiroiminodihydantoin; **d2Ih**: 5-carboxamido-5-formamido-2-iminohydantoin; **dIz**: 2,5-diaminoimidazolone; **dOz**: 2,2,4-triamino-2H-oxazol-5-one; **^Ox^dIa**: oxidizediminoallantoin; **(5′*R*/*S*)cdG**: (5′*R*/*S*)5′,8-cyclo-2′-deoxyguanosine, **R**: 2-deoxyribose; **P**: a internucleotide phosphodiester bond; * indicates the chiral carbon atom.

Sevilla et al. have shown that the radical cation, and therefore ^OXO^dG, are formed preferentially on the guanine located at the 5′-end position [33]. (This is in contrast to the DNA damage formation via radical activity like ●OH, which occurs in a random manner.) The formed G^●+^ can be converted to a neutral radical (G^●^), 2′-deoxyguanosine or, after reaction with water molecules, to 7,8-dihydro-8-oxo-2′-deoxyguanosine (^OXO^dG) [34,35]. It is well established that ^OXO^dG is one of the most abundant deoxyguanosine lesions, i.e., 14.62 ± 1.45 per 10^6^ DNA bases [36]. Furthermore, theoretically and experimentally, ^OXO^dG has been found to have a lower IP than that of the parent dG, with the following reduction potentials: 1.29 V for dG and 0.74 V for ^OXO^G [37,38]. In their experimental studies, Shuster et al. have shown that if ^OXO^dG appears in the ds-DNA structure during a one-electron oxidation process, then it becomes the final destination point of the migrated radical cation [28] (see Figure 2). The above indicates the possible protective role of 7,8-dihydro-8-oxo-2′-deoxyguanosine within the genome. It should be noted that ^OXO^dG exhibits low premutagenic ability when DNA damage response mechanisms are effective [39]. This lesion is removed from the genome by bifunctional OGG1 glycosylase (8-Oxo-Guanine Glycosylase 1), for example [40]. If this lesion goes unrecognized it can form a pair with adenine, which can lead to GC→TA transversion [41]. Fortunately, the adenine from the ^OXO^GA pair is recognized and removed by the MutY glycosylase (adenine DNA glycosylase) [42]. This system of two enzymes protects the genetic information against changes. Recently Barton et al. have shown that MutY utilizes the electron transfer through the double helix for DNA damage recognition, which makes this process extremely effective even for small numbers of proteins. (For details, please see references [43,44,45]). In brief, glycosylases which contain a [4Fe-4S]^2+^ cluster in their structure, like Muty, are able to scan dsDNA via the electron transfer between two “red-ox” proteins [46]. (It should be poined out that the iron–sulphur cluster is not required for their hydrolytic activity.) MutY recognises a damaged DNA base pair, i.e.,: ^OXO^G:dA, approximately 6–10 times faster than it does a native dA:dG base pair [47,48]. The protein electron communication process allows the whole genome to be scanned in a reasonable time and kept free of errors, even by a low number of glycosylase copies [49,50,51]. Schematically, the glycosylase (MutY) in its second oxidation state [4Fe-4S]^2+^ randomly binds to *ds*-DNA and subsequently is converted into [4Fe-4S]^3+^. If a released electron migrates, unhindered, through the double helix, a second Iron-Sulphur protein reduction can occur, releasing it from the ds-oligo. In contrast, if a DNA cation radical appears in the electron’s path, its migration is ended as a result of radical cation reduction. At this stage, a protein like Muty starts to migrate to the DNA damage location, where it recognizes and removes the damage. The protein containing [4Fe-4S] at the 3+ state binds to the double helix around 1000 times more strongly than its reduced form [52]. Therefore, the proposed mechanism should be preferred in the case when ^OXO^dG or a product of its further degradation exists in a one-electron oxidizing state [51] (Figure 3).

As mentioned previously, most studies have discussed DNA damage repair and induction in the context of a single isolated lesion. However, in the case of radiotherapy, chemotherapy, photodynamic or polytherapy, the number of DNA damage events increases, leading to the formation of clustered DNA lesions (CDLs) [53,54]. A CDL is defined as two or more DNA damage events per one or two helix turns. Therefore, to what extent other guanine lesions existing in the form of a multi-damage site interact with the charge transfer and protective role of ^OXO^dG remains unresolved. With reference to previous studies (Table 1), this paper presents a comparative theoretical analysis of the influence of a second lesion on the electronic properties of ^OXO^G:::C.

## 2. Results and Discussion

The critical point of DNA damage repair systems, and therefore the stability of genetic information, depends on the effective detection of lesions in the genome. This process must be much faster than the replication process and be active throughout the whole cell cycle [55]. The mechanism proposed by Barton, i.e., that glycosylases like MutYh utilize charge transfer for scanning ds-oligonucleotides, meets these criteria [43,56]. However, this process has only been investigated for isolated lesions. Here, a comparative analysis has been carried out to assess the effect of a second lesion in a CDL on the electronic properties of ^OXO^dG. Figure 2 presents the structures of selected DNA damage events as well as their mutual distribution within model ds-oligos, while the notation and corresponding ds-oligo sequences have been presented in Table 1. The results of all ds-oligo electronic properties presented in this paper were calculated at the M062x/6-31++G** level of theory in the aqueous phase, using the non-equilibrated and equilibrated modes of solvent–solute interaction. In detail, the discussed oligonucleotides are presented within the following references: [57,58,59,60,61,62,63], and are shown in Table 1. Comparing the adiabatic ionization potential (AIP) of the ds-pentamers shows that it decreases by ~0.20 eV after the appearance of ^OXO^dG in the double helix structure. This decrease occurs regardless of the presence of a second lesion except in the cases of *R* and *S* d2Ih when compared to the native *oligo-N*. The appearance of 2-aminohydantoin in the system almost completely negates the effect of ^OXO^dG. The difference in the oligo AIPs was found to be 0.01 eV and 0.08 eV for the *R* and *S* 2Ih diastereomers, respectively, in comparison to *oligo-N*. A similar effect was observed for the non-equilibrated and equilibrated vertical ionization potentials (VIP). Surprisingly, the appearance of a second ^OXO^dG caused slight increases in the VIP and AIP in comparison with *oligo-^O^G* (Table 1). The additional (extra) electron appearing in the system results in radical anion formation. As shown in Table 1 and Table S1 in the Supplementary Materials, the calculated vertical (VEA) and adiabatic electron affinity (AEA) for native *oligo-N* and oligonucleotides containing one or more lesions show only minimal differences, ranging from −0.08 to 0.06 eV, except for *oligo-Iz*, *oligo-Oz* and *oligo-*^OX^Ia, in which imidazolon, oxazolone or oxidized iminoalantoin were present as an additional lesion to ^OXO^dG. The presence of dIz and ^OX^dIa caused increases in the AEA of −1.35 and −0.95 eV, respectively; in contrast, Oz led to a decrease in AEA of 0.12 eV in comparison to native ds-DNA. It should be pointed out that the appearance of one or two ^OXO^dG moieties in the system did not change the electron affinity, leaving it at the same level as that assigned for an unmodified ds-pentamer, i.e., −1.90 eV. The above comparative analysis reveals that in almost all the 11 investigated ds-oligos, the presence of ^OXO^dG in the CDL structure did not significantly change the global electronic properties. The only exceptions were noted in the case of the AIPs for both diastereomers *R* and *S* of 2Ih, which almost completely negate the effect of ^OXO^dG, while dIz, dOz and ^OX^Ia had a significant influence on AEA. The last three can be perceived as four- and six-electron oxidation guanine products, whereas ^OXO^dG is only two-electron [64]. The above observations show the significant difference in electronic properties between the ds-DNA fragment containing a single lesion (^OXO^dG) and a multi-damage site in which ^OXO^dG is present as a 3′-end lesion (Figure 3). It is commonly accepted that 7,8-dihydro-8-oxo-2′-deoxyguanosine is the preferred spot for radical cation accumulation. Consequently, it has been suggested that it can protect the distal parts of ds-DNA against damage formation. This phenomenon can affect the charge transfer process and ultimately lead to changes in the protein–protein communication process. While the above is well documented in the case of single lesions as isolated base pairs, nucleotides, nucleosides or nucleo-bases, the properties of ^OXO^dG where additional lesions are present have been less thoroughly investigated [65]. It should be noted that the number of CDLs increases with higher radiation doses, chemotherapeutic intervention or combined anticancer therapy [54]. It can be postulated that the effectiveness of the above depends on slowing down the replication of cancer cells and reducing the recognition and repair processes of DNA damage [66,67].

Therefore, an increase in the number of “genome guards” (^OXO^dG) in undesired cell genomes can have the opposite effect to the one intended; i.e., the cancer cells may survive. To clarify these assumptions, a comparative analysis of the electronic parameters of the base pair settled at the position of ^OXO^G_4_C_2_ in ds-oligos (Figure 3) was conducted. The vertical and adiabatic ionization potential was considered in the equilibrated solvent–solute interaction mode [33]. The above is derived directly from the fact that the base pair settled in the middle part of the double helix is surrounded by water molecules only from the sides, while the flat aromatic ring of purines and pyrimidines interact with the proximal and distal base pairs via *π*–*π* interaction.

**Table 1:** The sequence (only the strands containing a DNA lesion have been shown) and corresponding oligonucleotide notation with DNA damage abundance per nucleotide (nt) or ^(a)^ guanosine (Gua) in cells, and vertical/adiabatic ionization potential (V/AIP) in eV calculated at the M06-2x/6-31++G** level of theory in the aqueous phase.

Name	Sequence	Abundance	VIP^NE^	VIP^EQ^	AIP
*oligo-N*	AGAGA [57]	0.64 × 10^9^ nt [36]	6.72	6.08	5.65
*oligo-^O^G*	AGA**^OXO^G**A [57]	14.6 per 10^6^ nt [68]	6.27	5.79	5.38
*oligo-^O^G^O^G*	A**^OXO^G**A**^O^G**A [57]		6.54	6.02	5.39
*oligo-^Fapy^G*	A**^Fapy^G**A**^O^**GA [58]	~8.0 per 10^6^ nt [68,69]	6.32	5.80	5.38
*oligo-Oz*	A**Oz**A**^**O**^**GA [59]	2–6 per 10^7^ Gua [38]	6.36	5.82	5.40
*oligo-Iz*	A**Iz**A^**O**^GA [59]	^(a)^ 2–6 per 10^7^ Gua [38]	6.25	5.78	5.37
*oligo-^OX^Ia*	A^OX^**Ia**A^**O**^GA [60]	Gh (^OX^Ia precursor) 1–7 per 10^8^ nt [70,71]	6.30	5.80	5.39
*oligo-(R)2Ih*	A***R*****2Ih**A^**O**^GA [61]	14.6 per 10^6^ nt. Similar to ^OXO^G [70,71]	6.56	5.94	5.57
*oligo-(S)2Ih*	A***S*****2Ih**A^**O**^GA [61]		6.53	5.90	5.50
*oligo-(R)cdG*	A***R*cdG**A^**O**^GA [62]	0.05 per 10^6^ nt [72]	6.32	5.82	5.40
*oligo-(S)cdG*	A***S*cdG**A^**O**^GA [63]	0.11 per 10^6^ nt [72]	6.37	5.86	5.39
*oligo-(R)Sp^ANTI^*	A***R*Sp**A^**O**^GA [63]	200 per 10^6^ Gua [73]	6.39	5.81	5.38
*oligo-(R)Sp^SYN^*	A***R*Sp**A^**O**^GA [63]		6.64	5.88	5.43
*oligo-(S)Sp^ANTI^*	A***S*Sp**A^**O**^GA [63]		6.35	5.88	5.37
*oligo-(S)Sp^SYN^*	dA***S*Sp**A^**O**^GA [63]		6.66	5.90	5.48
		Average	6.42	5.86	5.42
		Standard Deviation	0.14	0.07	0.06

NE—non-equilibrated, EQ—equilibrated solvent-solute interaction. ^(a)^ Iz as the precursor of Oz.

It is assumed that the latter is responsible for charge transfer via ds-DNA. Detailed analyses of charge and spin distribution within the base-pair ladder of ds-DNA after one-electron oxidation and radical cation formation were performed at the M06-2x/6-31++G** level of theory in the condensed phase according to Hirshfeld methodology [74]. Following the previous studies of Sevilla et al., the non-equilibrated and equilibrated modes of solvent–solute interaction have been considered an important factor involved in radical cation distribution [33]. This approach can be applied because in the aqueous phase, the whole structure of ds-DNA was submerged. As presented in Table 2, the charge and spin settle within ds-oligo, mainly on the ^OXO^G_4_:::C_2_ moiety, regardless of the presence of a second lesion in the CDL structure (except for oligo-^O^G^O^G). The following ranges were observed: 0.74–0.94 [au] for charge and 86–96 [%] for spin. Only in the case of oligo-^O^G^O^G, in which the second ^OXO^G was present (Table 1), were increases in charge and spin on ^OXO^G_4_:::C_2_ observed, along with progress in radical cation rearrangement (Table 2).

Ionization potential (IP) is defined as the ability to lose an electron [75]. According to the Franck–Condon principle, this process in the initial state occurs faster than nucleus relaxation [76]. Therefore, taking into account the charge transfer process and the system’s complexity, IP should be discussed in two modes: vertical and adiabatic. As previously mentioned, the solvent–solute can play a significant role. However, given the incomplete immersion of ^OXO^G_4_:::C_2_ in the condensed phase, only the VIP in the equilibrated mode was considered, as pointed out above. This parameter depends on the energy of the neutral (*E*_0_^0^) and cation ground state of the investigated molecule, which corresponds to energies *E*_0_^0^ and *E*_0_^+^, respectively. Therefore, the vertical IP and adiabatic IP should be different in the case of the ^OXO^G:::C model and ^OXO^G:::C extracted from the double helix. In the latter case, the discussed BP interacts by *π*–*π* stacking with distal and proximal base pairs, which can distort the expected structure. As shown in Table 2 in all the discussed cases, the positive charge and corresponding spin mainly accumulate on ^OXO^G_4_:::C_2_, which allows the calculation of the corresponding vertical and adiabatic ionization potentials.

As can be expected, the VIP and AIP values obtained for the ^OXO^G_4_C_2_ moiety were found to be lower than the value calculated for the corresponding G_4_C_2_ by 0.22 eV and 0.55 eV, respectively. It should be mentioned that according to previous studies, the G_2_C_2_ located at the 5′-end of *oligo-N* exhibits a lower IP than for G_4_C_2_, but it was still higher than the value found for the oxidized guanosine formed. Hence, it can be expected that the second lesion located close to 7,8-dihydro-8-oxo-2′-deoxyguanosine can affect its electronic properties. Detailed analysis reveals, however, something to the contrary: in all the discussed ds-oligos, ^OXO^G_4_C_2_ adopts VIPs ranging from 5.90 eV to 5.94 eV and AIPs from 5.51 eV to 5.57 eV. A significant fluctuation in vertical and adiabatic ionization potentials was observed for the DNA lesion at position X_2_C_4_, as shown in Table 3 (VIP [eV]: 5.93–7.69, AIP [eV]: 5.93–7.72). The above, taken together with the charge and spin distribution analysis, strongly supports Shuster’s hypothesis that if ^OXO^G appears in the ds-DNA structure, it becomes a radical cation sink and protects the remaining part of the double helix (genome) on the 3′ and 5′ ends [28]. Double-stranded DNA is not only subject to a direct one-electron oxidizing process forced by random external factors like hydroxyl radicals but can also be induced by photosensitizers (PS), as shown in Figure 1. The interaction of PS type I with UV gives rise to the formation of radical cations, which can migrate through ds-DNA [77,78]. As shown by Barton and Saito et al., the charge transfer can be observed over hundreds of angstroms until it settles at a predisposed point, usually G or clusters of Gs, due to their low IP values [30,45]. Therefore, the unaffected electron–hole transfer towards ^OXO^G can be deemed crucial for its protective role and for facilitating communication with proteins such as MutYh and ExoIII [79,80]. According to Marcus’ theory, charge transfer through a double helix can be described by several factors: the driving force (*ΔG*), activation (*E_a_*) and reorganization (*λ*) energies [81,82]. These are strongly dependent on the structures of reactants and products. Therefore, in the above context, the *π*–*π* interaction between ^OXO^GC and the base pair located at its 3′ or 5′-end with possible geometry distortion by other DNA lesions merits discussion [57].

**Figure 4:**
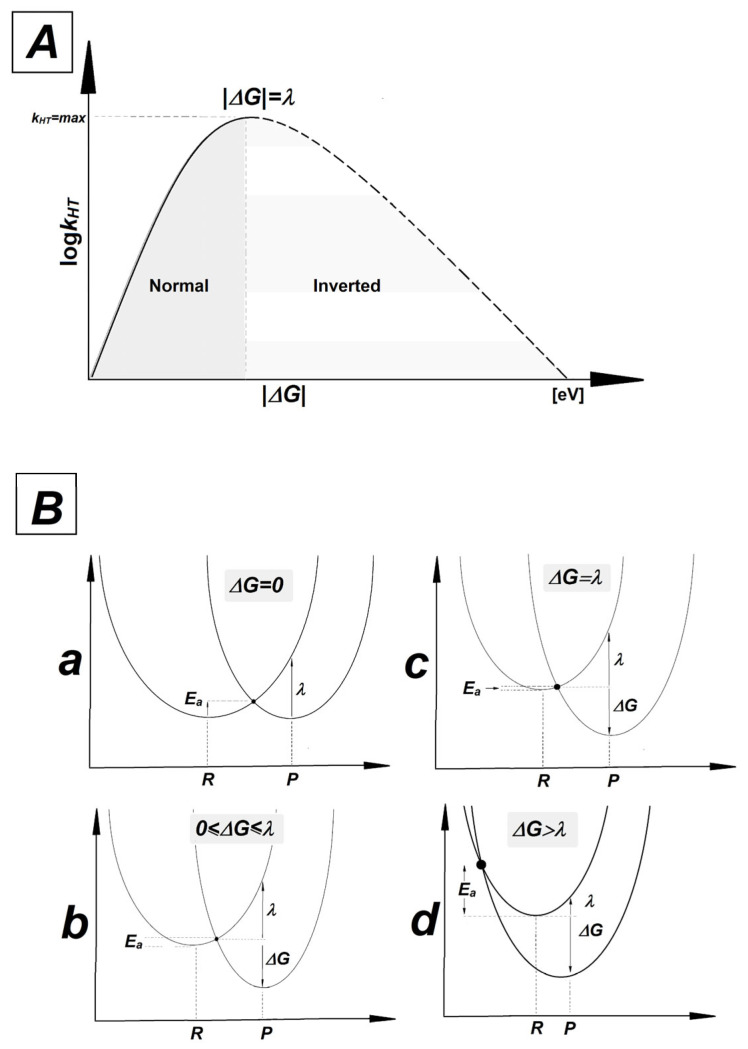
(**A**) The relationship between rate constant (*k_HT_*) and driving force (*ΔG*). The solid line represents the normal Marcus region and the dashed line represents the inverted region. A peak has been achieved for |*ΔG*| = *λ*. (**B**) Parabolas of the potential energy surfaces of reactant (R) and product (P) as a function of nuclear configuration: (**a**) isoergonic *ΔG* = 0, (**b**) Marcus normal region 0 ≤ *ΔG* ≤ *λ*, (**c**) for the maximum rate constant |*ΔG*| = *λ*, (**d**) Marcus inverted region |*ΔG*| > *λ*.

As presented in Table 4, the *ΔG*, *E_a_* and *λ* parameters calculated for hole transfers A_3_T_3_→^OXO^G_4_C_2_ and ^OXO^G_4_C_2_←A_5_T_1_ are very similar, but not identical. The average values in [eV] were found as follows: *ΔG*: −1.07, *E_a_*: 0.33, *λ*: 0.37 and *ΔG*: −1.12, *E_a_*: 0.37, *λ*: 0.38, respectively. For the neutral *oligo-N*, the discussed parameters were equal for the A_3_T_3_→G_4_C_2_ and G_4_C_2_←A_5_T_1_ charge transfers, i.e.: *ΔG*: −0.77, *E_a_*: 0.18, *λ*: 0.30. This can be directly attributed to the imperfection in the double helix geometry—the presence of a CDL—and the directionality of counter-rotating strands. This indicates that a lesion located towards the 5′-end of ^OXO^GC, beyond the single base pair, has less impact on the charge transfer between ^OXO^G_4_C_2_ and its neighboring base pair than expected, regardless of its structure. According to Marcus’ theory, the charge transfer between donor and acceptors in the case of the double helix can be endergonic (*ΔG* > 0), isoergonic (*ΔG* = 0) or exergonic (*ΔG* < 0) [83,84,85,86]. In the latter case, the maximum rate constant for the charge transfer in the Marcus normal region (satisfying the condition 0 ≤ |*ΔG*| ≤ *λ* and *E_a_* > 0) has been achieved for *λ* = |*ΔG*| with *E_a_* equal to zero. After reaching a peak and dropping into negative *ΔG* values with *ΔG* > *λ*, the activation energy increases and the rate constant of charge transfer (*k_HT_*) decreases. This part of the parabola is the so-called Marcus inverted region. *k_HT_* is mathematically described as *k_HT_* = A × exp^(−*Ea*/RT)^ [86]. A graphical representation of the relationship between *k_HT_*, *ΔG*, *λ* and *E_a_* is shown in Figure 4.

A comparative analysis of parameters presented in Table 4 reveals that in all cases, the electron–hole transfer settles in the Marcus inverted region. For all the discussed cases, the absolute values of the driving force are higher than reorganization energy, with moderate values of *E_a_*. The quadratic relationship between driving force and activation energy given by the following equation, *E_a_* = *λ*/4(1 + *ΔG*/*λ*)^2^, indicates that as exergonicity increases, *k_HT_* decreases. It should be pointed out that the *E_a_* observed for the charge transfer process between the GC and AT base pairs within *oligo-N* was almost two times lower than that noted for the corresponding ^OXO^GC in the discussed ds-oligos. The above indicates the significant role played by 7,8-dihydro-8-oxo-2′-deoxyguanosine in hole migration, revealing it as a suitable point for radical cation settling, regardless of the presence of other kinds of lesion.

## 3. Materials and Methods—Applied Computational Strategy

All theoretical calculations presented in this article were carried out according to previous descriptions [47,48,49,50,51,52,53]: the starting geometries of bi-stranded oligonucleotides were built using BIOVIA Discovery Studio Visualizer v20.1.0.19295 software [87] and denoted as presented in Table 1 [57,58,59,60,61,62,63]. In all cases the negative charges of the phosphate groups were neutralized by the addition of protons. The structure of all discussed *oligonucleotides* were optimized in condensed phase using the ONIOM (our own N-layered Integrated Molecular Orbital and Molecular Mechanics) strategy [88], i.e.: nucleobases have been described on M06-2X/D95** level of theory while on the sugar-phosphate ladder by M06-2X/sto-3G [89,90,91,92]. The M06-2X functional with augmented polarized valence double-ζ basis set 6-31++G** was used for energy calculations. Additionally, for optimized geometries in each cases, charge and spin analyses were achieved using the Hirshfeld methodology at the M06-2X/6-31++G** level of theory [74]. The electronic properties of molecules were calculated as described previously [93,94]. The solvation–solute interaction was investigated in both non-equilibrium (NE) and equilibrated (EQ) modes [95]. All the above calculations were performed using the Gaussian G16 (version C.01) software suite [96].

## 4. Conclusions

The activity of various harmful factors can lead to different DNA damage formations, which, left unrepaired, give rise to mutations and/or contribute to ageing and cancerogenesis. While isolated DNA lesions have been well investigated [97], the electronic properties of clustered lesions and their influence on the charge transfer rate have received far less attention. The comparative analysis of DFT studies reveals that the presence of another dG lesion in close proximity to ^OXO^G_4_C_2_ had no influence on its vertical and adiabatic ionization potentials, with the average values found being as follows: 5.92 ± 0.06 eV and 5.55 ± 0.15 eV, respectively.

In all the short ds-oligos discussed in the article, ^OXO^G_4_C_2_ becomes the point where the radical cation settles. The positive charge and spin mainly accumulate on the ^OXO^G_4_C_2_ base pair, irrespective of the vertical^NE/EQ^ or adiabatic cation state.

The values calculated for *ΔG*, *E_a_* and *λ* indicate that in all the discussed cases, the electron–hole transfers A_3_T_3_→G_4_C_2_ and G_4_C_2_←A_5_T_1_ settle in the Marcus inverted region and are resistant to a second DNA lesion appearing in close proximity. The comparative analysis of DFT theoretical studies presented in this paper indicates that the ^OXO^GC base pair acts as a sink for radical cations migrating through the double helix, regardless of the presence of other 2′-deoxyguanosine lesions in the structure of clustered DNA damage. This is in good agreement with the results of previous experimental studies conducted for isolated lesions.

## Figures and Tables

**Table 2:** The Hirsfeld charge and spin concentration (distribution) on a ^OXO^G_4_:::C_2_ base pair in [au] and [%] respectively as part of a CDL located in vertical and adiabatic radical cation modes of the discussed ds-oligo, calculated at the M06-2x/6-31++G** level of theory in the aqueous phase.

ds-Oligo	Radical Cation Mode of Ds-Oligo			Ref.
	Vertical^NE^	Vertical^EQ^	Adiabatic	
	Charge/Spin	Charge/Spin	Charge/Spin	
*oligo-^O^G*	0.80/89	0.85/91	0.83/93	[57]
*oligo-^O^G^O^G*	0.38/39	0.39/41	0.73/83	
*oligo-(R)cdG*	0.82/90	0.87/87	0.87/84	[62]
*oligo-(S)cdG*	0.88/91	0.92/93	0.86/92	
*oligo-(R)Sp^ANTI^*	0.76/87	0.80/80	0.80/90	[64]
*oligo-(R)Sp^SYN^*	0.90/82	0.91/85	0.93/85	
*oligo-(S)Sp^ANTI^*	0.85/90	0.89/91	0.80/90	
*oligo-(S)Sp^SYN^*	0.93/86	0.94/89	0.94/86	
*oligo-Iz*	0.75/87	0.79/90	0.83/93	[45]
*oligo-Oz*	0.81/98	0.89/92	0.83/92	
*oligo-^ox^Ia*	0.76/88	0.81/90	0.83/93	[60]
*oligo-^Fapy^G*	0.79/86	0.84/90	0.84/90	[58]
*oligo-(R)2Ih*	0.85/95	0.88/96	0.88/96	[61]
*soligo-(S)2Ih*	0.85/96	0.88/96	0.89/96	
Av.	0.79/86	0.83/87	0.85/90	
SD	0.14/14	0.14/14	0.05/4.0	

NE—non-equilibrated, EQ—equilibrated solvent solute interaction. Av.—average value, SD—standard deviation.

**Table 3:** The electronic properties in [eV] of ^OXO^G:::C and X_2_::C_4_ base pairs isolated from ds-DNA containing a second lesion in the CDL system, obtained at the M06-2x/6-31++G** level of theory in the aqueous phase. The following ds-DNA structure was taken into consideration: d[A_1_**X_2_**A_3_**^OXO^G_4_**A_5_]*d[T_1_C_2_T_3_C_4_T_5_], X = a second DNA lesion.

Ds-Oligo	d[A_1_X_2_A_3_^OXO^G_4_A_5_]*d[T_1_C_2_T_3_C_4_T_5_]					
	X	X_2_:::C_4_		^OXO^dG_4_:::C_2_		
		VIP^EQ^	AIP	VIP^EQ^	AIP	Ref.
*oligo-^O^G*	G	6.17	6.16	5.91	5.56	[57]
*oligo-^O^G ^O^G*	^OXO^G	5.93	5.93	5.91	5.56	
*oligo-^Fapy^G*	^Fapy^G	6.17	6.16	5.90	5.56	[58]
*oligo-Iz*	Iz	7.04	7.03	5.94	5.51	[59]
*oligo-Oz*	Oz	7.01	7.03	5.91	5.56	
*oligo-^OX^Ia*	^OX^Ia	7.07	7.06	5.94	5.52	[60]
*oligo-(R)2Ih*	(*R*)2Ih	7.03	7.02	5.94	5.54	[61]
*oligo-(S)2Ih*	(*S*)2Ih	6.93	6.94	5.93	5.53	
*oligo-(R)cdG*	(*R*)cdG	6.15	6.19	5.93	5.56	[62]
*oligo-(S)cdG*	(*S*)cdG	6.14	6.18	5.93	5.57	
*oligo-(R)Sp^ANTI^*	(*R*)Sp*^ANTI^*	7.66	7.64	5.93	5.53	[63]
*oligo-(R)Sp^SYN^*	(*R*)Sp*^SYN^*	6.93	6.91	5.91	5.54	
*oligo-(S)Sp^ANTI^*	(*S*)Sp*^ANTI^*	6.90	6.91	5.90	5.56	
*oligo-(S)Sp^SYN^*	(*S*)Sp*^SYN^*	7.69	7.72	5.90	5.56	
Av.		6.77	6.78	5.92	5.55	
SD		0.57	0.60	0.06	0.15	
*oligo-N*	X_2_, ^OXO^G_4_ = G	6.13	5.83	6.13	6.11	[47]

**Table 4:** Charge transfer parameters calculated at the m062x/6-31++G** level of theory in the aqueous phase and given in [eV] of permissible transfers between OXOG4C2 and A3T3 or A5T1 base pairs.

Ds-Oligo	d[A1X2A3^OXO^G_4_A_5_]*d[T_1_C_2_T_3_C_4_T_5_]						Ref.
	A_3_T_3_→^OXO^G_4_C_2_			^OXO^G_4_C_2_←A_5_T_5_			
	*ΔG*	*E_a_*	*λ*	*ΔG*	*E_a_*	*λ*	
*oligo-^O^G*	−1.09	0.40	0.35	−1.12	0.30	0.41	[57]
*oligo-^O^G ^O^G*	−1.09	0.39	0.35	−1.12	0.32	0.40	
*oligo-^Fapy^G*	−1.07	0.40	0.34	−1.13	0.34	0.39	[58]
*oligo-Iz*	−1.15	0.28	0.37	−1.18	0.52	0.36	[59]
*oligo-Oz*	−1.07	0.33	0.44	−1.12	0.39	0.34	
*oligo-^OX^Ia*	−1.10	0.27	0.42	−1.16	0.46	0.35	[60]
*oligo-(R)2Ih*	−1.10	0.32	0.38	−1.08	0.35	0.37	[61]
*oligo-(S)2Ih*	−1.09	0.32	0.39	−1.09	0.35	0.36	
*oligo-(R)cdG*	−0.99	0.22	0.40	−1.11	0.36	0.38	[62]
*oligo-(S)cdG*	−1.11	0.33	0.39	−1.11	0.29	0.41	
*oligo-(R)Sp^ANTI^*	−1.09	0.29	0.41	−1.15	0.34	0.41	[53]
*oligo-(R)Sp^SYN^*	−1.02	0.47	0.29	−1.13	0.38	0.37	
*oligo-(S)Sp^ANTI^*	−0.94	0.26	0.34	−1.12	0.36	0.38	
*oligo-(S)Sp^SYN^*	−1.05	0.40	0.33	−1.11	0.44	0.34	
Av.	−1.07	0.33	0.37	−1.12	0.37	0.38	
SD	0.05	0.07	0.04	0.03	0.06	0.02	
*oligo-N*	−0.77	0.18	0.30	−0.77	0.18	0.30	[57]

*ΔG*—driving force, *λ*—reorganisation energy, *E_a_*—activation energy. Arrows indicate the direction of charge migration.

## Supplementary Materials

The following supporting information can be downloaded at: https://www.mdpi.com/article/10.3390/molecules29163756/s1, Table S1. The sequence (only the strand containing a DNA lesion was shown) and corresponding oligonucleotides’ notation with DNA damage and vertical/adiabatic ionization potential and electron affinity (V/AIP) (V/AEA) in [eV] calculated at M06-2x/6-31++G** level of theory in aqueous phase. NE—non-equilibrated, EQ—equilibrated solvent–solute interaction. References [57,58,59,60,61,62,63] are cited in the Supplementary Materials.
