# Supplementary material for: The Influence of 2′-Deoxyguanosine Lesions on the Electronic Properties of OXOG:::C Base Pairs in Ds-DNA: A Comparative Analysis of Theoretical Studies
Source: Molecules. 2024 Aug 8;29(16):3756. doi: 10.3390/molecules29163756 (PMC11357419; doi:10.3390/molecules29163756)
Supplement: Supplementary file 1 [file molecules-29-03756-s001.zip › molecules-3092247-supplementary.pdf]

Supplementary Materials

# The Influence of 2′-Deoxyguanosine Lesions on the Electronic Properties of $^{OXO}$G:::C Base Pairs in Ds-DNA: A Comparative Analysis of Theoretical Studies

Boleslaw T. Karwowski

**Table S1**. The sequence (only the strand contained DNA lesion was shown) and corresponding oligonucleotides notation with DNA damage and vertical/adiabatic ionisation potential and electron affinity (V/AIP) (V/AEA) in [eV] calculated at M06-2x/6-31++G** level of theory in aqueous phase. NE- non-equilibrated, EQ- equilibrated solvent-solute interaction.

| Name | sequence | VIP$^{NE}$ | VIP$^{EQ}$ | AIP | VEA$^{NE}$ | VEA$^{EQ}$ | AEA |
|---|---|---|---|---|---|---|---|
| *oligo-N* | AGAGA[57] | 6.72 | 6.08 | 5.65 | -0.84 | -1.58 | -2.09 |
| *oligo-$^{o}$G* | AGA$^{OXO}$GA[57] | 6.27 | 5.79 | 5.38 | -0.85 | -1.43 | -2.04 |
| *oligo-$^{o}$G$^{o}$G* | A$^{OXO}$GA$^{O}$GA[57] | 6.54 | 6.02 | 5.39 | -0.86 | -1.59 | -2.09 |
| *oligo-$^{Fapy}$G* | A$^{Fapy}$GA$^{O}$GA[58] | 6.32 | 5.80 | 5.38 | -0.90 | -1.41 | -2.09 |
| *oligo-Oz* | AOzA$^{O}$GA[59] | 6.36 | 5.82 | 5.40 | -0.95 | -1.49 | -2.06 |
| *oligo-Iz* | AIzA$^{O}$GA[59] | 6.25 | 5.78 | 5.37 | -1.53 | -2.22 | -2.83 |
| *oligo-$^{OX}$Ia* | AIaA$^{O}$GA[60] | 6.30 | 5.80 | 5.39 | -2.42 | -3.08 | -3.59 |
| *oligo-*(*R*)*2Ih* | A*R*2IhA$^{O}$GA[61] | 6.56 | 5.94 | 5.57 | -1.00 | -1.68 | -2.00 |
| *oligo-*(*S*)*2Ih* | A*S*2IhA$^{O}$GA[61] | 6.53 | 5.90 | 5.50 | -1.07 | -1.40 | -2.09 |
| *oligo-*(*R*)*cdG* | A*R*cdGA$^{O}$GA[62] | 6.32 | 5.82 | 5.40 | -0.99 | -1.33 | -2.11 |
| *oligo-*(*S*)*cdG* | A*S*cdGA$^{O}$GA[62] | 6.37 | 5.86 | 5.39 | -1.01 | -1.60 | -2.12 |
| *oligo-*(*R*)*Sp$^{ANTI}$* | A*R*SpA$^{O}$GA[63] | 6.39 | 5.81 | 5.38 | -0.62 | -1.37 | -1.94 |
| *oligo-*(*R*)*Sp$^{SYN}$* | A*R*SpA$^{O}$GA[63] | 6.64 | 5.88 | 5.43 | -0.69 | -1.38 | -1.87 |
| *oligo-(S)Sp$^{ANTI}$* | A*S*SpA$^{O}$GA[63] | 6.35 | 5.88 | 5.37 | -0.68 | -1.44 | -1.97 |
| *oligo-(S)Sp$^{SYN}$* | d[A*S*SpA$^{O}$GA[63] | 6.66 | 5.90 | 5.48 | -0.67 | -1.34 | -1.34 |
| | | 6.42 | 5.86 | 5.42 | -1.01 | -1.62 | -2.15 |
| | | 0.14 | 0.07 | 0.06 | 0.45 | 0.46 | 0.49 |


57. Karwowski, B. How Clustered DNA Damage Can Change the Electronic Properties of ds-DNA , Differences between GAG , GAOXOG ,OXOGAOXOG. *Biomolecules* **2023**, *13*, 1–21.
58. Karwowski, B.T. Fapy dG in the Shadow of OXO dG — A Theoretical Study of Clustered DNA Lesions. *Int. J. Mol. Sci.* **2023**, *24*, 1–14.
59. Karwowski, B.T. The influence of clustered DNA damage containing Iz/Oz and 2 OXOdG on charge transfer through the double helix: a theoretical 3 study. *Molecules* **2024**, 1–17.
60. Karwowski, B.T. The influence of oxidized imino-allantoin , in the presence of OXO G , on double helix charge transfer : a theoretical approach. *IJMS* **2024**, 1–16.
61. Karwowski, B.T. The 2Ih and OXOG Proximity Consequences on Charge Transfer through ds -DNA : Theoretical Studies of Clustered DNA Damage. *Molecules* **2023**, *28*, 1–16.
62. Karwowski, B.T. The Influence of 5',8-Cyclo-2'-Deoxyguanosine on ds-DNA Charge Transfer Depends on Its Diastereomeric Form: A Theoretical Study. *Antioxidants* **2023**, *881*, 1–16, doi:10.3390/antiox12040881.
63. Karwowski, B.T. The Influence of Spirodi(Iminohydantoin) on Charge Transfer through ds-DNA Containing 8-OXO-dG: A Theoretical Approach. *Int. J. Mol. Sci.* **2023**, *24*, doi:10.3390/ijms24108570.
